# Hybrid FES-robot cooperative control of ambulatory gait rehabilitation exoskeleton

**DOI:** 10.1186/1743-0003-11-27

**Published:** 2014-03-04

**Authors:** Antonio J del-Ama, Ángel Gil-Agudo, José L Pons, Juan C Moreno

**Affiliations:** 1 Biomechanics and Technical Aids Unit, National Hospital for Spinal Cord Injury, SESCAM, Toledo, Spain; 2 Bioengineering Group, Spanish National Research Council, Madrid, Spain

## Abstract

Robotic and functional electrical stimulation (FES) approaches are used for rehabilitation of walking impairment of spinal cord injured individuals. Although devices are commercially available, there are still issues that remain to be solved. Control of hybrid exoskeletons aims at blending robotic exoskeletons and electrical stimulation to overcome the drawbacks of each approach while preserving their advantages. Hybrid actuation and control have a considerable potential for walking rehabilitation but there is a need of novel control strategies of hybrid systems that adequately manage the balance between FES and robotic controllers. Combination of FES and robotic control is a challenging issue, due to the non-linear behavior of muscle under stimulation and the lack of developments in the field of hybrid control. In this article, a cooperative control strategy of a hybrid exoskeleton is presented. This strategy is designed to overcome the main disadvantages of muscular stimulation: electromechanical delay and change in muscle performance over time, and to balance muscular and robotic actuation during walking.

Experimental results in healthy subjects show the ability of the hybrid FES-robot cooperative control to balance power contribution between exoskeleton and muscle stimulation. The robotic exoskeleton decreases assistance while adequate knee kinematics are guaranteed. A new technique to monitor muscle performance is employed, which allows to estimate muscle fatigue and implement muscle fatigue management strategies. Kinesis is therefore the first ambulatory hybrid exoskeleton that can effectively balance robotic and FES actuation during walking. This represents a new opportunity to implement new rehabilitation interventions to induce locomotor activity in patients with paraplegia.

Acronym list: 10mWT: ten meters walking test; 6MWT: six minutes walking test; FSM: finite-state machine; t-FSM: time-domain FSM; c-FSM: cycle-domain FSM; FES: functional electrical stimulation; HKAFO: hip-knee-ankle-foot orthosis; ILC: iterative error-based learning control; MFE: muscle fatigue estimator; NILC: Normalized stimulation output from ILC controller; PID: Proportional-Integral-derivative Control; PW: Stimulation pulse width; QUEST: Quebec User Evaluation of Satisfaction with assistive Technology; SCI: Spinal cord injury; TTI: torque-time integral; VAS: Visual Analog Scale.

## Introduction

Spinal cord injury (SCI), due to the resulting functional loss, is one of the most devastating clinical conditions with negative consequences on independence. Several assistive technologies are available for functional compensation of gait as well as for restoring walking function. The use of hip-knee-ankle-foot orthosis (HKAFO) to provide lower limb joint support dates back to the 1950s, allowing swing-through mobility, with the use of walkers or crutches. The mobility achieved with this device is aesthetically poor and requires higher metabolic energy expenditure, which limits its use for daily ambulation. The introduction of reciprocating mechanisms for hip joint improved mechanical efficiency of orthotic gait, thus reducing the energy cost of ambulation [1]. However, the required energetic cost and upper extremity loading during such aided ambulation are still excessive [2].

The main rationale behind such high physical demand of either reciprocating or fixed orthoses, is that the energy required for ambulation comes primarily from the upper extremity, which in turn leads low efficiency walking patterns. Active orthoses, or robotic wearable exoskeletons (hereinafter only exoskeletons), by adding actuators at the orthotic joint, provide an external source of controlled joint power. Many active exoskeletons have been developed for gait restoration, with much variation in the actuator and sensing technologies. However, whilst there are some commercially available devices, like the ReWalk or Ekso, the technology is not mature enough to produce unlimited community ambulation yet [3,4].

An alternative technology for generating joint movement is Functional Electrical Stimulation (FES) of weak or paralyzed muscles of a SCI person during functional activities [5]. FES has been widely explored as a means of gait compensation in people with SCI [6], which provides both physiological and psychological benefits to the impaired user [6,7]. However, early appearance of muscle fatigue [8-10] and difficult control of joint trajectories [11,12] are limiting factors for its widespread use as rehabilitation or functional compensation of walking. There have been many attempts to improve gait performance and decrease energy expenditure by combining FES with passive or reciprocating orthoses, but such hybrid orthoses have only provided reduced improvements in energy costs and walking velocity [13].

A further hybrid approach attempts to combine the FES and active exoskeletons to overcome the drawbacks of each approach, while preserving their advantages. A review on hybrid exoskeletons, [14], defined “hybrid exoskeletons” as systems that aim to compensate and/or rehabilitate gait in activities of daily living by means of delivering and controlling power to the lower limb joints, in which the net joint power results from the combination of muscle activation with FES and electromechanical actuation. Hybrid exoskeletons were classified in two main groups as to how they control the power delivered to the joint: 1) semi-active hybrid exoskeletons, and 2) fully active hybrid exoskeletons.

Semi-active hybrid exoskeletons are those that dissipate power at the joint, which is produced by the stimulated muscles and gravitational forces acting over the lower limb. Precise control of joint trajectory is achieved by brakes or clutches placed at the exoskeleton joints. These systems consider the FES as an intermittent power source, and are low weight and energy efficient systems.

However, although stimulation demand is minimized, muscular fatigue due to FES would eventually appear, especially in case of neurological impairment, and the system’s efficacy would decrease. On the other hand, fully active hybrid exoskeletons are those that can both dissipate and deliver power to the joint. This way, the lack of muscular response in neurologically injured individuals and the muscle fatigue due to the stimulation can be compensated. However fully-active systems are bulky and energetically inefficient.

Regarding the control of the hybrid exoskeleton, open-loop and closed-loop stimulation control approaches were found. Open-loop control strategies generally have a pre-programmed stimulation pattern, which is sequenced through the detection of gait events (e.g. floor contact or swing). Joint trajectory control of exoskeleton’s actuators has been proposed, with a feedback controller of trajectory or interaction joint torque [15-18]. A representative example is the hybrid exoskeleton developed by Kobetic and Marsolais [16], designed over the basis of an implanted FES system with 16 channels. It is able to provide variable control of the hip and knee joints. The implanted FES system generates walking from a pre-programmed stimulation pattern, while the robotic exoskeleton detects walking states and transitions, providing control of gait events.

However, this FES control strategy does not allow to react to changes in muscular performance through stimulation modulation. Furthermore, open loop FES control does not allow to optimize the balance between muscle-elicited and exoskeleton power during movement. Monitoring of muscular performance is critical in semi-active exoskeletons, where joint power generation for movement relies only on the stimulated muscle.

Closed-loop control of FES relies on feedback of indirect measures of muscle performance. One of those measures is the joint movement generated by the muscle under stimulation, which has been implemented in [19] to modulate stimulation timing. Another indirect measure is the physical interaction between the leg moved by the stimulation and the attached exoskeleton [20]. Stimulation amplitude is controlled by comparing the interaction torque with a torque pattern previously recorded in healthy subjects. With closed-loop FES control, it is possible to automatically compensate a reduction in muscle performance by increasing a stimulation parameter (pulse width, amplitude, train frequency) to generate the desired joint torque or position. However, muscle extenuation is likely to occur under this approach, and therefore, explicit recognition of muscle fatigue is a requirement, together with a strategy to manage muscle fatigue.

The stimulation control strategy implemented in [21] can be regarded as a combination between open loop and closed loop approaches. There, indirect measures of low stimulation performance (through trajectory error) or excessive stimulation (through brake actuation) are averaged during the gait cycle and weighted together, leading to a constant value that scales the stimulation pattern of the next step. Thus, the stimulation is in a way modulated in a step-by-step basis. However, the exoskeleton actuators in this approach are semi-active, and thus cannot provide an active strategy to circumvent muscle fatigue, leading to an insufficient joint trajectory control [15,21].

While hybrid actuation and control have a considerable potential for rehabilitation of locomotion, novel control strategies are still required that implement a real balance between FES and robotic controllers, beyond than synchronized application of both torque sources with independent controllers, and exploiting the inherent advantages of each modality of actuation. To our best knowledge, theoretical frameworks for combining FES and robot technologies have been proposed to optimize FES and robot actuation over the user [22,23], but no experimental results have been reported. Combination of FES and robotic control is still a challenging issue, due to the non-linear behavior of stimulated muscles and the relatively short spectrum of development in the field of control of hybrid exoskeletons.

The objective of this article is to, firstly, present a novel cooperative control strategy of a hybrid exoskeleton for gait rehabilitation of people with SCI and, secondly, to technically validate the control approach in a group of healthy subjects. Validation experiments to verify system’s design and control approach are crucial before performing tests on the target population. Thus, in this article we present results from an experimental study with a group of healthy subjects. The study seeks to verify the following hypotheses: 1) the control approach is able to balance FES and robotic control of movement under a therapeutic approach, and 2) muscular performance can be monitored to manage muscle fatigue to eventually increase treatment time.

The *Material and methods* section of this article is organized as follows. In subsection *Kinesis: a hybrid lower limb exoskeleton for SCI rehabilitation*, a KAFO-type exoskeleton is presented, and with it the design of its high level control strategy, conceived to deliver the cooperative behavior with the electrical muscle stimulation. In subsection *Stimulator controller* the description of the closed-loop FES control strategy is presented, followed by a description of the proposed on-line estimator of muscle fatigue (subsection *Muscle fatigue estimator*). In the next subsection *Cooperative approach*, the description of the cooperative control approach is built over the previous components. These sections constitute the presentation of the cooperative control approach. Subsection *Evaluation with healthy subjects* presents the experimental protocol that has been executed in order to technically validate the hybrid control approach.

## Material and methods

### Kinesis: a hybrid lower limb exoskeleton for SCI rehabilitation

**Figure 1:**
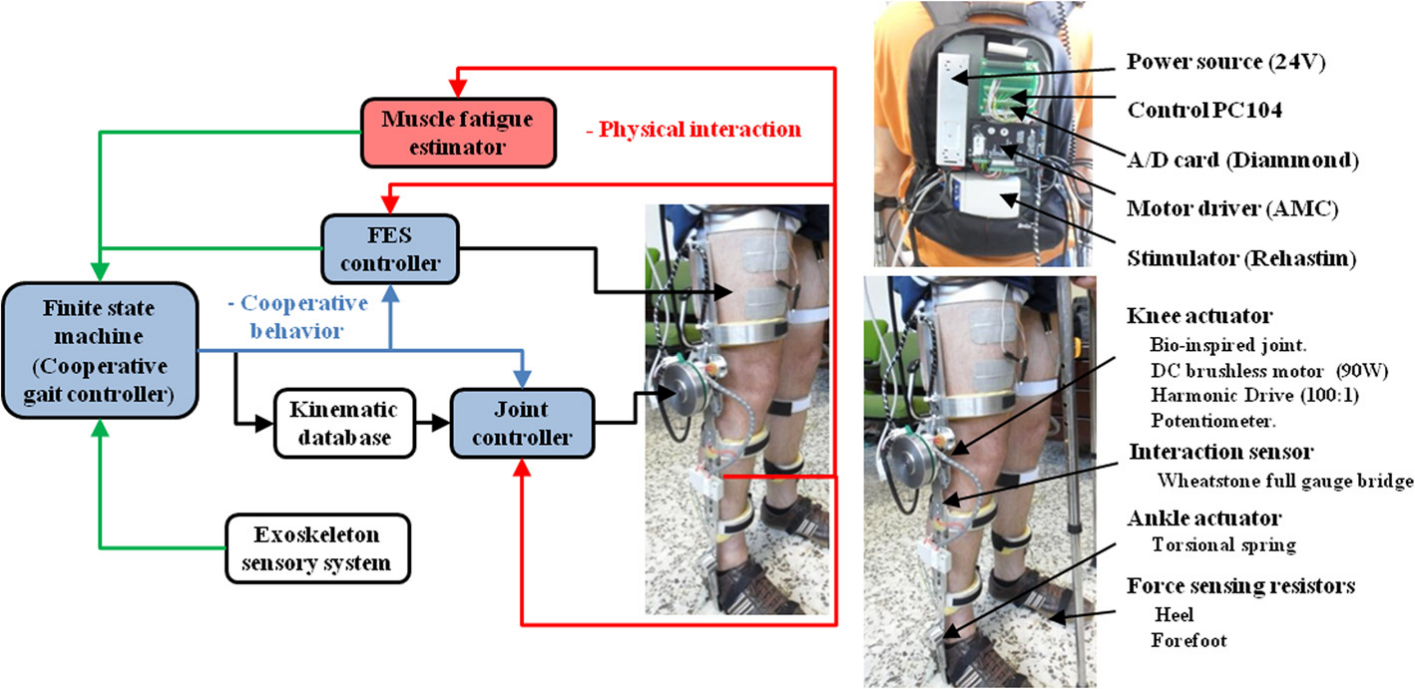
**Kinesis hybrid exoskeleton and cooperative control approach.** Left: High level cooperative controller. Top right: backpack containing control electronics, motor drivers, stimulator, PC and power source. Bottom right: Mechanical description and sensor placement.

There are various examples of hybrid exoskeletons for compensation of walking described in the literature [14]. The Kinesis system presented here has been designed to compensate gait in patients with low level of SCI and has been presented elsewhere [24] (Figure 1, right). Kinesis has been developed to test a hybrid rehabilitation approach for SCI individuals whose lesion is referred to as *Conus Medularis*[25]. This type of lesion is characterized by paralysis of muscles driving the knee and ankle joints, while hip flexors (*psoas*) are preserved.

Kinesis is a knee-ankle-foot exoskeleton, equipped with an active actuator the knee (a Maxon DC flat motor, 90W and a Harmonic-Drive 100:1 gear), a passive elastic actuator at the ankle, force sensing resistors for monitoring floor contact and user commands, potentiometers for measuring knee position and a full Wheatstone bridge to measure interaction torque. The controller was implemented in a PC-104 embebed computer using the xPC target environment (The MathWorks, Inc., Natick, MA). Kinesis has a PC-controlled stimulator (Rehastim, Hasomed GmbH) which delivers biphasic current-controlled rectangular pulses. Rehastim can be pulse width and current controlled in real time. Further information on Kinesis design is available in [24,26].

The high-level control approach to achieve a cooperative behavior is shown in Figure 1. The controller comprises four main components: 1) a robotic or joint controller, 2) a FES controller, 3) a muscle fatigue estimator (MFE), and 4) a finite-state machine (FSM), that coordinates the FES and joint controllers. In the following sections a description of the four components is given. The cooperative approach is then described in the next subsection.

### Knee joint control

In order to realize compliant actuation for one degree of freedom (knee joint), impedance control is employed to set joint stiffness as a function of interaction torque. This strategy enables the optimization of the muscle-induced movement rather than constraining the final movement to a fixed trajectory. Several research groups are recognizing these limitations of position controlled exoskeletons, implementing control schemes to provide a more flexible robot, adaptable to the functional capabilities of the user [27]. By following this concept, the joint controller of Kinesis applies a torque field around a reference knee joint trajectory during overground walking. In this way, mechanical behavior varies from constrained trajectory control to unhindered motion, allowing to adapt Kinesis compliance to muscular FES performance.

(1) $$\tau =\text{Kk}\cdot \left(\theta_{\text{pattern}}-\theta_{\text{actual}}\right)+\text{Ck}\cdot \frac{\Delta \left(\theta_{\text{pattern}}-\theta_{\text{actual}}\right)}{\mathrm{\Delta t}}$$

**Figure 2:**
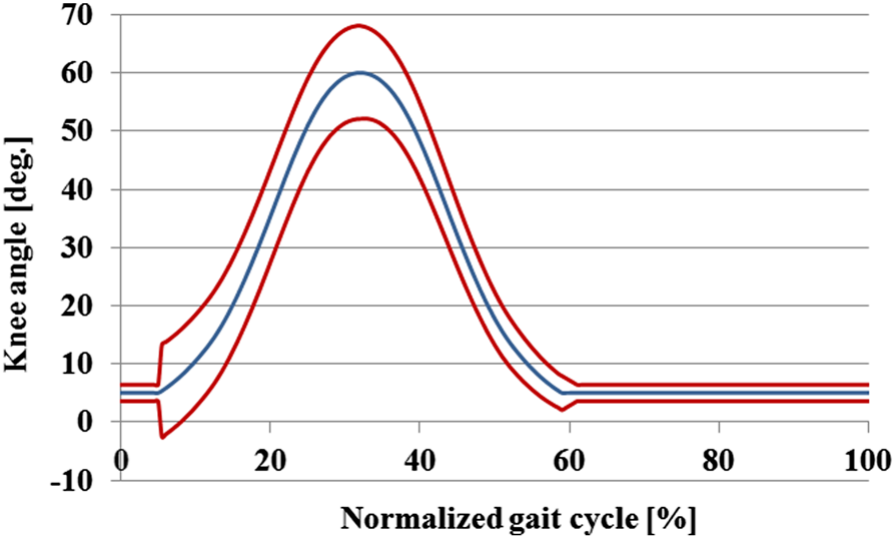
**Schematic of the stiffness control approach of Kinesis.** Blue curve is the kinematic pattern stored in controller memory. Red is a representation of the kinematic range generated by the stiffness control approach.

A first order torque field is imposed around the knee joint trajectory, therefore the torque imposed by the exoskeleton is a function of the deviation of the knee trajectory from a given reference pattern (equation 1). The kinematic pattern for the swing phase was extracted from a normative database available at our laboratory, comprised by biomechanical data of walking of healthy subjects. Data corresponding to slow walking speed was selected. The kinematic pattern for the stance phase was reduced to a constant value, as explained below (Figure 2).

The stiffness *Kk* of the torque field, in our approach, is modulated depending on gait events as follows. During stance, it is paramount to provide joint support to avoid knee collapse, therefore a high stiffness torque field is needed. During the swing phase the joint stiffness must be reduced, to allow for the contribution of stimulated muscles and passive dynamics to swing and move over a range of speeds. This is achieved by reducing the support of the exoskeleton through the torque field. In order to illustrate the concept, Figure 2 depicts the reference kinematic pattern in blue and the boundaries of the torque field in red. As initial criteria for choosing the value of the stiffness for both gait phases, we have followed the same approach developed in a previous work [28], where the knee stiffness during walking is modeled as linear with different values for stance and swing. The stiffness of the torque field, in our approach, varies from 6 Nm/deg for stance, up to 0 Nm/deg during swing if knee trajectory is fully developed by the stimulated muscle, and there is no need of exoskeleton support. Damping of the torque field *Ck* was tuned by trial and error to improve controller stability.

**Figure 3:**
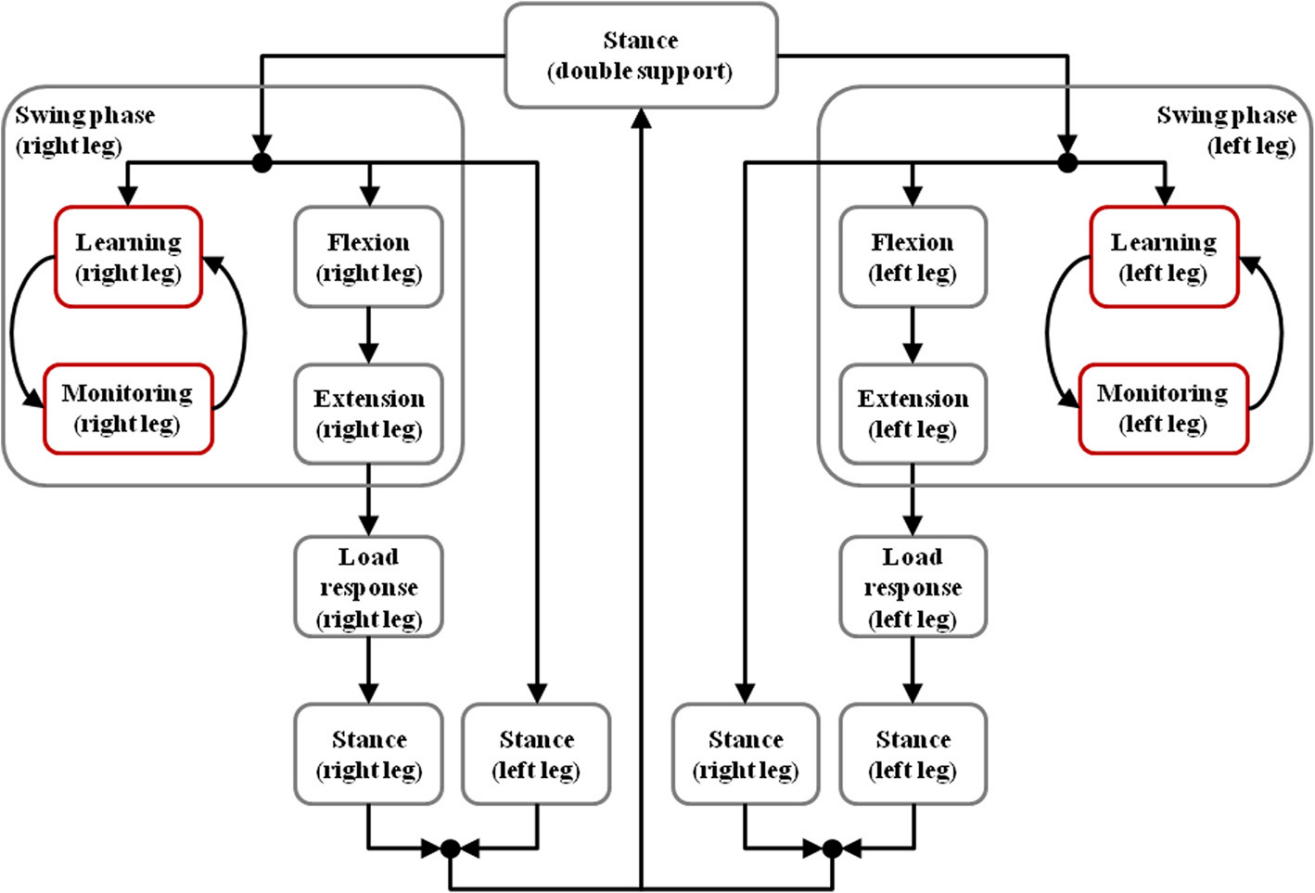
**Kinesis finite-state machine.** The swing states of the t-FSM contains the operation of the c-FSM (labeled in red). Conditions for transition between states are not shown for simplicity.

Admittance control was chosen for implementing the control strategy, in order to achieve a stable behavior during the stance phase. The admittance control scheme is designed over a velocity control loop integrated within the electrical motor driver (American Motion Controls). Detection of gait events is performed by a finite state machine (t-FSM^a^) that gathers information from the sensors. The design of our time domain FSM is similar to other reported FSMs for ambulatory exoskeletons, take for instance [16,28] (Figure 3).

### Muscle fatigue estimator

As stated in the Introduction, one of the major drawbacks of electrically elicited movement by means of FES is the development of muscle fatigue. Research efforts have been directed to analyze pulse and train configurations to achieve a more physiological stimulation, thus generating more muscle force per pulse train and delaying the appearance of muscle fatigue [8,29-31]. However, muscle fatigue would eventually develop, and in our opinion very little attention has been paid to investigate strategies that may manage muscle fatigue during repetitive functional tasks.

Although muscle fatigue models can be found in the literature, a criteria for early detection of muscle fatigue has not been proposed. Recording the evoked electromyographical signal (eEMG) of the stimulated muscle as indicator of muscle performance has been proposed, but the correlation between eEMG and muscle fatigue is still controversial [32-34]. In addition, the complexity of recording and processing eEMG regarding muscle fatigue still remains challenging, requiring specific and custom-made equipment for rejecting artifacts from the stimulation [33]. Early detection of muscle fatigue would allow investigating novel methods to manage it, in line with the diverse methods for FES-generated gait already reported.

In this sense, we have recently proposed a method for estimate fatigue onset of knee flexor muscles, based on monitoring changes on the generated force [35,36]. This method relies on measuring the torque-time integral (TTI) generated by the stimulation, and monitoring the time-evolution of the TTI. We found out that, under constant stimulation parameters, namely unchanged pulse configuration and train duration, decay in TTI of 19% is due to muscle fatigue. Therefore, the muscle fatigue estimator (MFE) implemented in Kinesis measures limb-exoskeleton interaction torque and calculates the TTI during the swing phase, as presented in [24], to estimate muscle fatigue.

Since the fatigue criteria need the stimulation parameters to be constant in order to monitor the time-evolution of the TTI, we have implemented a two-steps algorithm in the controller. During the first step, the stimulation patterns are optimized, under a Iterative Learning Control (ILC) approach. Once the stimulation patterns are found, this is, when the ILC has converged, these patterns are hold constant for the following swing cycles, and then the MFE can be applied as described. Once muscle fatigue is detected, the cooperative controller changes system’s behavior. Details regarding the implementation of this algorithm are provided in section *Cooperative approach*, where the steps of the algorithm are defined as *learning*, and *monitoring* respectively.

### Stimulator controller

The use of FES to restore walking ability in SCI is known since the early works done by Kantrowitz [37]. Ever since, several researchers have been developing technology and control strategies to achieve walking restoration with the use of FES [38-41]. In open-loop control of FES, stimulation patterns are manually selected and then sequenced, usually triggered by a hand switch or automated with a gait sensor. However, the human body is a highly complex musculoskeletal system and, although some systems have a considerable number of implanted electrodes, the gait patterns generated do not resemble typical normal gait of healthy individuals.

Closed-loop control of FES has also been proposed in the literature. In particular, adaptative feedback control [11], model based control [42], rule-based control [43,44], iterative Proportional-Integral-Derivative (PID) control [45], sliding mode control [46,47], model predictive control [48], neural networks and fuzzy control [11,49,50] iterative error-based learning control (ILC) [51-54], have been proposed. In spite of all these efforts to control FES-mediated gait, accurate movement control is still difficult to perform due to existing parameter variations (e.g., muscle fatigue), inherent time-variance, time-delay, and strong non-linearities present in the neuromuscular-skeletal system, in muscle activation relation, muscle dynamics, and skeletal dynamics [55].

Implementation of most of those closed-loop control approaches in hybrid ambulatory exoskeletons is difficult, given its computational burden and the lengthy controller tuning processes, which would make the set-up time unaffordable for the use in clinical practice. In addition, swing and stance phases of gait during stimulated (or hybrid) walking have inherent differences that can be exploited when choosing a controller. The swing phase can be determined by joint trajectory and time. Conversely, the stance phase must be determined based on a stability criterion prior to the initiation of a new step. Moreover, several uncertainties arise due to limb orientation at heel contact and whole body orientation or balance. Thus, in our approach we have implemented a dual closed-loop FES controller, in which knee extensor muscles are controlled by a PID controller and the flexor muscles are controlled by an iterative error-based learning controller. Both controllers are fed-back with information on physical interaction between the limb and the exoskeleton to modulate knee flexor and extensor muscles stimulation. The control task is to minimize the interaction torque through modulation of electrical stimulus pulse width.

PID control of extensor muscles is an easy and effective method to avoid knee joint collapse during stance or double support. Although the variety of uncertainties and non-linearities are not adequately managed by PID controllers, we have assumed that quadriceps muscles will not be overstimulated, as the robotic exoskeleton would provide joint support during stance. Thus, quadriceps are stimulated when the knee is not fully extended at stance, while support is guaranteed by robotic exoskeleton. Potential knee joint collapse is measured in Kinesis as an increase in interaction torque during flexion, which is feed to the PID controller resulting in an increase in quadriceps stimulation and thus extending knee joint. In section *Evaluation with healthy subjects* the method for adjusting the PID constants is described.

Swing phase is a time- and trajectory- defined task in which the human leg and the exoskeleton mutually interact. The swinging motion of the leg during this phase fits exactly within the ILC setting for periodic and cyclical over a finite interval with resetting between trials. By incorporating error information into the control for subsequent iterations, high performance can be achieved with low transient tracking error in spite of large model uncertainty and repeating disturbances [56]. Applications of ILC for FES control have been demonstrated in [51,52], following the general form [56]:

(2) $$\left\{u_{n,j+1}\right\}=\;[\;F]\cdot \left[\left\{u_{n,j}\right\}+\;[\;L]\cdot \left\{e_{n,j}\right\}\right]$$

In this equation, {*u*_*n*,*j*+1_} is the FES control vector to be applied in the next step *j*+1, where n is the number of time frames that compose the swing phase (note that the kinematic pattern during swing phase is time-defined). It is calculated from the control vector applied within step *j*, modified by the error produced by this control vector {*e*_*n*,*j*_} multiplied by a learning constant matrix [ *L*], and both affected by a forgetting constant matrix [ *F*] [56].

The control task assigned to the FES controller is to minimize interaction torque between leg and exoskeleton. During swing Kinesis drives user’s leg following the kinematic pattern stored. When the leg does not move along with the trajectory pattern, Kinesis measures the interaction forces resulting from weight and inertia of the leg. Therefore this interaction is forwarded to the ILC controller to generate a FES control signal for next step that aims to minimize the interaction. Note that within this approach, interaction can have two complementary sources: the lack of torque delivered by the user to move the leg during swing and the inability of the human joint to follow the reference kinematic pattern.

Equation 2 updates each *n*-*th* component of the control vector {*u*_*n*,*j*+1_} with information of the same time-frame interaction torque at step *j*, which is originated by the effects of control component *u*_*j*_ and the system’s behavior at this time-frame, which depends on the effects of previous control signals over the leg. Given the considerable delay between stimulation onset and torque generation, we have modified the algorithm, and a non-casual learning feature was introduced, to give to the ILC the ability to take into account errors that the control signal produces in future samples. This non-causal learning feature is introduced in the learning matrix [ *L*] as a semi-Gaussian window centered in the sample *j* which module is the learning factor of the ILC. Length and module of the Gaussian window, which forms the learning matrix [ *L*], and forgetting constant matrix were set by manual tunning in experiments with several healthy subjects.

The output of both PID and ILC controllers is a control signal that modulates the stimulation pulse width to regulate muscle force for extension and flexion respectively, while pulse amplitude and frequency are held constant. These parameters are fed to the Rehastim stimulator, which applies stimulation to the extensor and flexor muscle groups.

### Cooperative approach

This section presents the methodology followed for the three previously presented controllers (exoskeleton knee joint, electrical stimulation and muscle fatigue) to work in a cooperative fashion. Figure 1 left depicts physical interaction (red line), cooperative control commands (blue line) and controller outputs (black line). The cooperative behavior of Kinesis allows to obtain adequate and personalized stimulation patterns, estimating muscle fatigue and reducing robotic assistance during overground assisted gait. This approach intends to give priority to the use of artificially stimulated muscles to generate leg movements.

In order to implement and test such performance, we have designed a FSM that operates in the domain of the gait cycle (c-FSM, Figure 3, right), one for each leg, during swing phase, coordinated with the t-FSM that operates in the time domain (presented in section *Kinesis: a hybrid lower limb exoskeleton for SCI rehabilitation* (Figure 3, left)). The t-FSM coordinates the left and right c-FSM by broadcasting cycle events: once a leg enters in swing state, a new step event is broadcasted to the respective cycle-domain FSM, either left or right. Each c-FSM has two states: *learning state* and *monitoring state*.

*Learning state* is the default state when the user commands the first step. Within this state, the ILC controller iterates, as showed in section *Stimulator controller*. In the first step the stimulation output from the ILC is zero and the system’s goal is to drive the leg during swing. Then torque field stiffness is high enough to produce a position control of knee trajectory. This produces an interaction torque resulting from the mass and inertia of the leg, which is fed as error signal to the ILC for the first iteration. In subsequent steps, resulting from ILC stimulation, the interaction torque decreases, fed as error signal to the ILC for further iterations. By calculating the gradient of the stimulation output time-integral (see NILC definition in *Data analysis* subsection), the ILC convergence is assumed when this gradient is lower than 5%. Therefore the *monitoring state* is entered. Within this state, the last control vector output from ILC is stored in memory and repeated as stimulation pattern during the next steps, and the ILC algorithm is stopped. Then the MFE monitors the TTI, and Kinesis modulates its assistance in a cycle-by-cycle basis, decreasing the knee torque field stiffness following an approach similar to [45]. This decrease in assistance is done while a knee flexion objective of 60 degrees is achieved. This is, Kinesis decreases the torque stiffness up to the minimum value that allows a minimum knee flexion angle of 60 degrees. Once muscle fatigue is estimated by the MFE, by an increase of 19% of TTI, a muscle fatigue management approach can be deployed [36]. In our approach, we change stimulation train parameters^b^ for delaying muscle fatigue. This change on stimulation configuration is required for a new iteration period for the ILC to learn the new system state.

### Safety

Several safety measures were implemented in Kinesis. Ankle and knee exoskeleton joints were equipped with mechanical stops in the physiological limits of motion. In addition to this, the admittance controller of the knee joint was programmed with a software limit at maximum and minimum positions. In case of exceeding these limits, the state machine executes the locking of the motor shaft, then moving back to a default safe knee position. A third software safety measure consists on the limitation of the maximum output torque demanded to the motor. An equivalent safe strategy was implemented in the stimulator controller to set safety limits for pulse width and amplitude modulation. Finally, a mechanical safety button was deployed to physically disconnect the energy supply of the entire hardware system. Safety tests were conducted to verify the adequate actuation and response of the safety measures before actually moving to the evaluation phase, described in the following section.

### Evaluation with healthy subjects

Four healthy volunteers participated in the protocol to test the performance of the Kinesis cooperative control approach (Age 30.5 ± 1.7, weight 77.0 ± 5.7 Kg height 1.8 ± 0.1 m). The protocol was designed to be applicable for involvement of incomplete spinal cord injured subjects in a further stage. The time to complete each walking test was set to 6 minutes, similarly to the 6 minutes walking test (6mWT). The reason was to set a suitable time for the walking test and to obtain information regarding walking function of persons with SCI. The time needed to walk the first 10 meters was also recorded, also known as 10 meters walking test (10MWT). We choose these tests extensively used in clinical practice for measuring walking performance of persons with SCI [57]. The experimental protocol also included a Visual Analog Scale (VAS) about user fatigue and comfort, and the QUEST (Quebec User Evaluation of Satisfaction with assistive Technology, [58]) items related with system performance.

Participants signed an informed consent before the initiation of the experiments, including the use of images and video recorded during the experiments. The experimental procedure was approved by the ethics Review Board of the National Hospital for Spinal Cord Injury. Three electrodes (Alexgaard, Pals-platinum) were placed over the motor points of *Vastus Lateralis*, *Rectus Femoris* and *Vastus Medialis* knee extension muscles, and two electrodes over the motor points of *Semitendinosus* and *Biceps Femoris* knee flexion muscles [59]. Then, a muscular warming period with electrical stimulation was carried out during 5 minutes. Stimulation parameters were: pulse width 200 *μ*s, frequency 8 Hz, train 14 seconds, duty cycle 43% and the amplitude set to the contraction threshold.

After this warming period, pulse width was set to 450 *μ*s and train frequency to 70 Hz, and an iterative search of the pain threshold, by increasing pulse amplitude, was performed. As the maximum pulse width of the FES controller was limited to 450 *μ*s, we aimed to obtain the maximum tolerable amplitude at that pulse width, the user pain threshold. Then, the participant worn the exoskeleton and a tuning procedure of the FES-PID controller was carried out. PID tuning was performed by using the heuristic frequency response method developed by Ziegler and Nichols [60].

After a relaxing period of 10 minutes, the walking test began. Given that the main objective was to test Kinesis cooperative control approach, participants were instructed to simulate the functional ability of the target population: “walk passively avoiding voluntary movements of both knee and ankle, bending to one side to lift the heel, and drag the hip”. The walking experiment consisted of walking with Kinesis during 6 minutes in straight line assisted by a walker. The skin under electrodes was inspected after the experiment, as well as exoskeleton and cabling conditions.

## Data analysis

Time needed to walk 10 meters and distance covered in 6 minutes from all participants were group averaged. Mean and standard deviation for VAS, 10mWT and 6MWT were obtained, and mode and range were calculated for QUEST scores. Kinesis performance was assessed in terms of actual knee angle, torque interaction, stimulator control output and torque field stiffness.

The normalized average stimulation output for knee extensor and flexor muscles (acronym NILC for flexor muscles) were calculated during the swing and stance phases respectively. This normalized average was calculated integrating the stimulator output during the walking phase (for swing and stance separately), and dividing the result by the maximum stimulation output theoretically achievable, which corresponds to a 450 *μs* saturated output for the entire walking phase. This normalized stimulation output gives a representative value ∈ [ 0,1] where 0 means no stimulation during the entire phase, and 1 means a constant, saturated stimulation output of 450 *μs* for the entire walking phase.

Energy delivered by Kinesis actuator was estimated with the electrical power consumed by the motor, disregarding mechanical efficiency. We assumed that mechanical losses are low and approximately constant. A correlation analysis was performed between estimated delivered energy during swing and TTI for both legs of all participants (Spearman’s Rho correlation test, p-value < 0.05).

## Results

Operation of Kinesis was well tolerated: users felt comfortable and no dangerous situations were reported. After the experiment, electrodes were removed and the skin revealed slight erythema that disappeared within 10 next minutes. No adverse effects were reported during the experiments. In one experiment, data from one leg were lost due to a connection malfunction.

**Figure 4:**
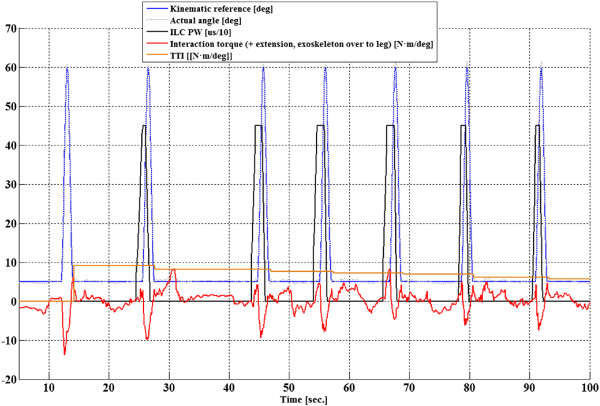
**Representative results in time domain from participant 3.** Data corresponds to the first steps of a walking trial. Representative data from left leg of participant 3 during the first 100 seconds of the experiment. Knee reference angle (blue), actual knee angle (dotted gray curve), user-exoskeleton interaction torque (red curve), stimulation pulse width (PW) output from the ILC controller (ILC PW, black curve, scaled by a factor of 10), and TTI (brown curve. TTI curve is updated after completing the swing phase). Note the decrease on interaction torque during swing phase, due to increasing muscle contribution to the movement during this phase.

**Figure 5:**
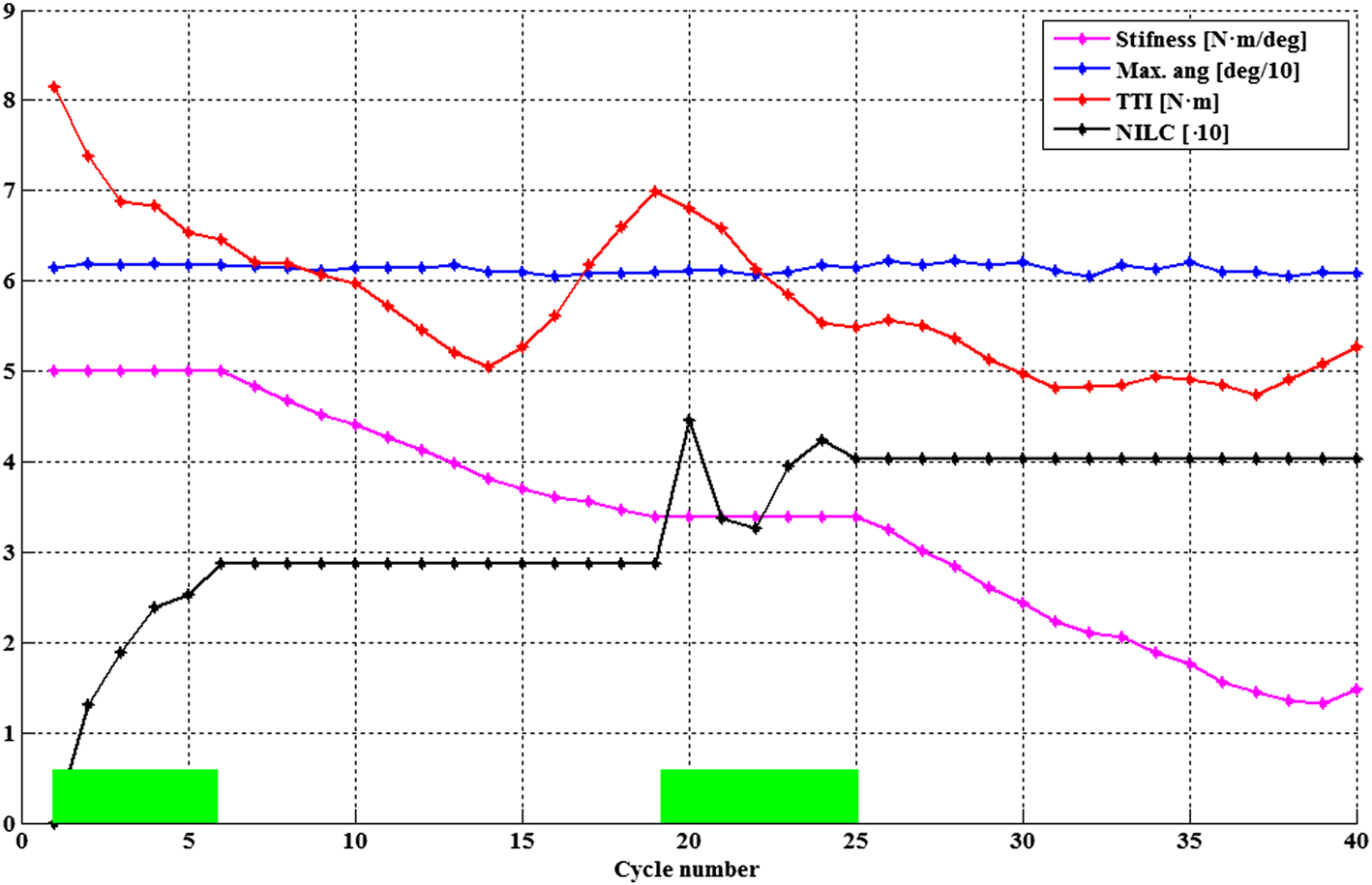
**Representative results in cycle domain from participant 3.** Data corresponds to the entire walking trial. X-axis is cycle number. TTI (red), maximum knee flexion angle (blue), exoskeleton stiffness (pink), normalized stimulation output (NILC, black) for each step are shown. Green boxes show *learning state* active, otherwise means *monitoring state* active.

**Figure 6:**
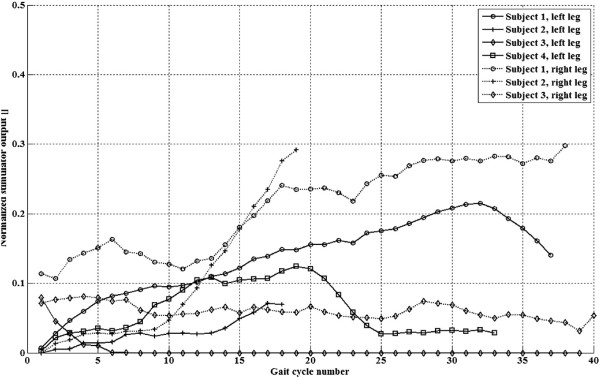
**Normalized quadriceps stimulation pulse width during stance.** Data from both legs of all four participants. Note that in one experiment, data from one leg were lost due to connection malfunction.

**Figure 7:**
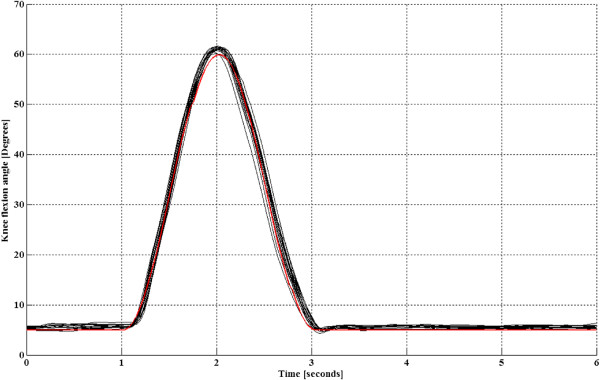
**Representative example of actual knee kinematics from subject 3, left leg.** Representative data from right leg of one participant during the entire experiment. Knee reference angle is superimposed in red. As noticed, actual kinematics remains closer to the reference.

**Figure 8:**
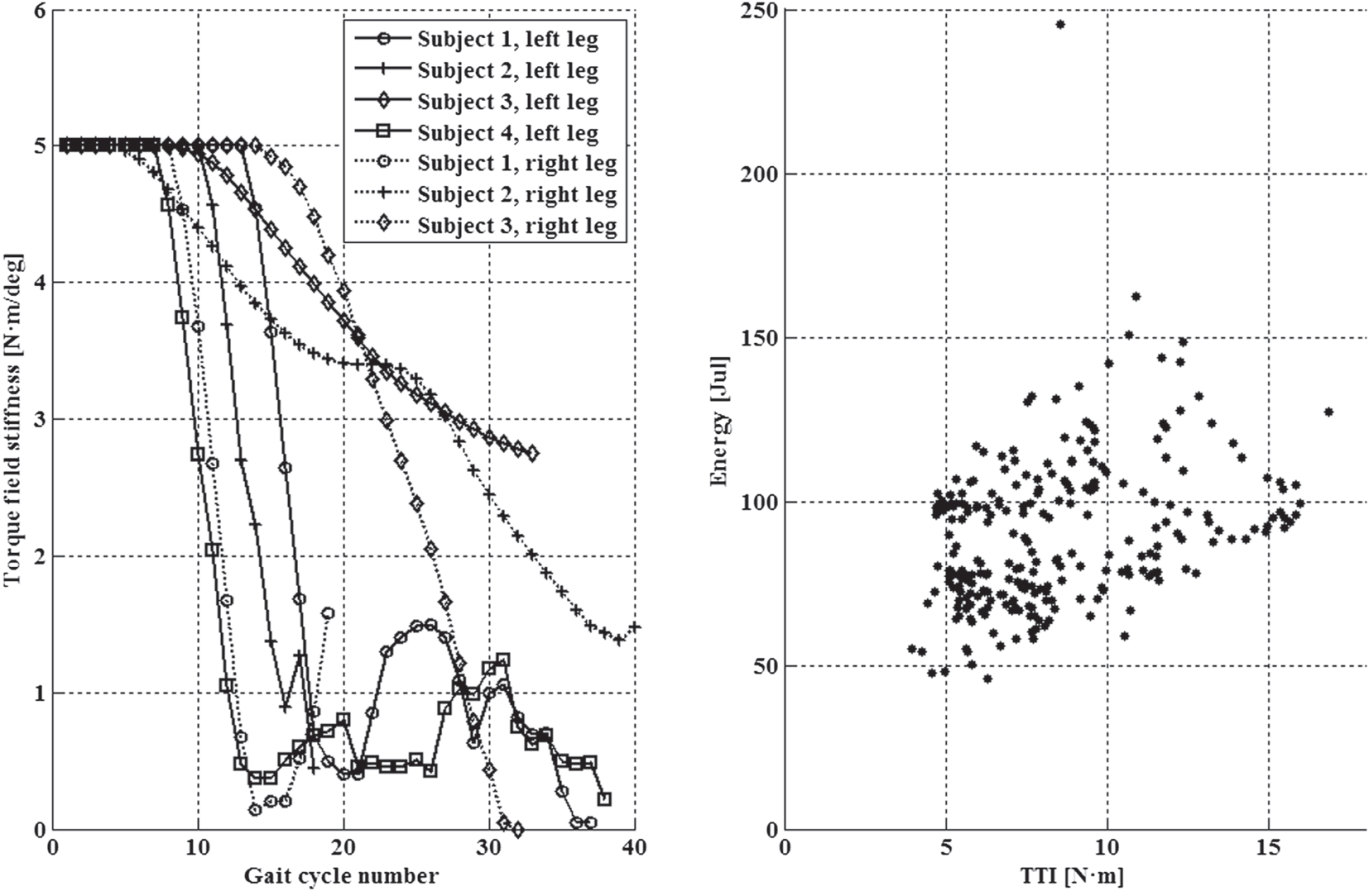
**Kinesis compliance adaptation.** Left: Kk progress for both legs of all participants. Rigth: energy delivered by Kinesis actuator during swing phase VS TTI (R = 0.34, p < 0.001 Spearman’s Rho correlation test). Data from both legs of all four participants. Note that in one experiment, data from one leg were lost due to connection malfunction.

To illustrate our findings, experiment results from one participant are shown in Figures 4 and 5. Time evolution of the main controlled variables with Kinesis during the first steps of an experiment for left leg of participant number 3 is shown in Figure 4. Knee trajectory, interaction torque, TTI during swing phase, and ILC stimulation output. Knee kinematic pattern during stance was set to 5 degrees (0 is full extension), as it is a mean value achieved by the knee during stance in healthy, accelerated, walking. This angle was set to avoid hyperflexion during stance. It can be observed that a knee position near 5 degrees was successfully maintained, with a small compliant deviation (maximum range of knee deviation for all experiments between 2 to 8 degrees). Figure 6 shows the normalized quadriceps stimulation output during stance for both legs of all participants. It can be observed that stimulation during stance was in average small, below 30%. Figure 7 shows the actual normalized knee angle of an experiment for left of participant 3. Note that the reduction in the displayed stiffness of Kinesis (see Figure 8 left, participant 3, left leg) does not impact on the actual knee trajectory, while toe clearance is guaranteed by achieving 60 degrees of knee flexion.

Transitions amongst stance and swing were smooth during the experiments and no jerky movements were noticed (Figure 7). Swing knee trajectory of first steps was trajectory controlled (Figure 4, blue and light blue curves) while the ILC was iterating. Interaction torque during swing shows a progressive reduction in the peak flexor torque when comparing subsequent steps (Figure 4, red curve). This reduction and modulation can be related to the stimulation effect during swing (Figure 4, black curve). This reduction can be better noticed when looking at the TTI during swing in Figure 4, red step-like curve, or in Figure 5, where the main controlled variables for same participant and leg are represented in cycle domain, for the entire experiment. Normalized stimulation output from the ILC (NILC) was calculated as the envelope of actual ILC control signal (black curve of Figure 4) relative to a maximum envelope that represents the maximum stimulation during swing phase.

Figure 6 presents the progress of the *learning state*, from the start until cycle number 6. During this state, ILC stimulation is gradually increased, TTI decreases, and the maximum flexion angle is maintained above 60 degrees. From cycle 4 to 6, a stabilization in ILC output can be observed. In cycle 6 the relative change in NILC is lower than 5%, therefore convergence is assumed and the system enters in *monitoring state*. Within this state stiffness for the swing phase is progressively decreased, while actual knee trajectory and maximum flexion angle are maintained (Figure 7). Therefore the corrective actions of the robotic exoskeleton over the knee are also decreasing. A further TTI decrease is observed (Figure 5, cycles 7 to 14). Although stimulation is held constant, this can be understood as an effect due to accommodation of the stimulated muscle. Besides, users could voluntary activate muscles during movement. After cycle 15 a gradual increase on TTI is observed. This is due to a decrease in muscle performance, indicating muscle fatigue appearance. After overcoming the fatigue threshold in cycle 19, the stimulation parameters are changed. This change in stimulation parameters and muscle dynamics requires a new iteration period, therefore Kinesis enters in *learning state*. In cycle 26 a further ILC convergence is estimated, and Kinesis enters in *monitoring state*. Number of steps for convergence was 11,0 ± 3,3 in average for both legs, and fatigue was detected 19.4 ± 1,5 steps after the beginning of the walking trial.

Figure 8 shows how the control of knee torque field stiffness operates for both legs of all participants. Note that in some cases, Kinesis was able to reduce Kk to 0 N ·m/deg, indicating that the robotic exoskeleton does not provide assistance to drive the knee during swing, only the stimulated muscles. A correlation analysis between the energy delivered by the exoskeleton during swing phase and the TTI, shows that a reduction in TTI reflects a significative reduction in the energy delivered by the exoskeleton (Figure 8).

**Figure 9:**
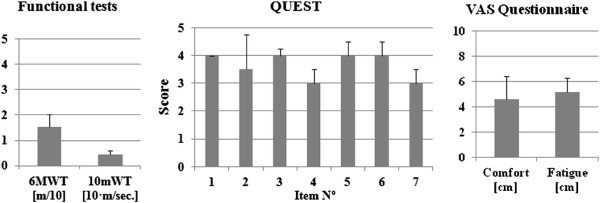
**Functional results of the testing group.** Group results. Left: 10 meters test. Center: QUEST score. Right: pain and comfort VAS score.

Figure 9 shows group results of functional walking test and questionnaire scores: QUEST and VAS score for pain and fatigue. Average data for the testing group were 0.44 ± 0.14 m/sec. for the 10mWT, and 15.4 ± 5.0 meters for the 6MWT. QUEST items were scored in the middle of the scale (type Likert from 0 to 5), except for items 4 and 7. Comfort (4.6 ± 1.8 cm) and fatigue (5.2 ± 1.1 cm) perceived by the users was also set at the middle of the VAS scale.

## Discussion

Firstly, our analysis has shown that the proposed control approach is able to balance neuroprosthetic and robotic contributions with a therapeutic approach to induce locomotor activity. This has been confirmed by means of the correlation analysis of energetic contribution by the robot and performance of artificially activated muscles. However, the efficiency of this control design needs to be further investigated with respect to the therapeutic application. Secondly, our analysis has shown that the muscle performance in hybrid FES-robot control of gait in a group of healthy subjects can be monitored and quantified in terms of human-robot interaction. The proposed MFE is able to manage stimulation performance for iterative learning and monitoring FES-driven torque to manage the effect of muscle fatigue. A considerable period of training (typically several weeks or even months) with electrical stimulation is required to apply such neuroprosthetic solution [38,40,61] or hybrid exoskeletons [15,16,21] for gait-related tasks in SCI, mainly due to changes in muscular characteristics after paralysis. With the proposed MFE it is possible to shorten the stimulation training period significantly, combining part of the training period with the hybrid walking therapy. The MFE can potentially detect muscle extenuation, thus the stimulation can be disconnected while continuing walking therapy. However, the proposed approach, assumes a uniform effect of fatigue for the involved stimulated muscles around the knee joint. Monitoring the activation of each stimulated muscle independently would represent a more precise estimation of fatigue. Nevertheless, the methodology proposed here aims to manage muscle fatigue due to FES within this unique hybrid actuation context, specifically designed for this application. Our method is therefore not a solution for muscle fatigue management, but particular technique that appears to be effective in sustaining average generated joint torques in hybrid actuation context. On the other hand, we did not implement a more physiological stimulation approach (like multi-electrode stimulation) due to the complexity to control the force generation for functional purposes.

Closed loop control of FES is implemented in few hybrid ambulatory exoskeletons [20], and some have a sort of semi-closed loop control [19,21]. To the best of our knowledge, results on proper closed-loop control of FES have not been reported before. Through quantification of TTI during swing, Kinesis gives an objective measure about stimulation effectiveness of flexor muscles, a highly demanding stimulation. Extensor muscles are only stimulated when knee flexes during stance. Although blocking the knee through the exoskeleton eliminates the need of stimulating the extensor muscle, Kinesis PID FES control allows for a more physiological control of the knee during stance. Results showed that stimulation was reduced 80% compared with an ON-OFF stimulation sustained for the entire stance phase. Although semi-active hybrid exoskeletons achieve greater reduction in quadriceps stimulation (e.g. 89% reduction in [21]), our stimulation approach provides a more physiological stimulation, related to joint bending during stance. However, the inability of measuring voluntary muscle activation limits the interpretation of these data. Although the participants were instructed to avoid activation of the leg muscles, verification of this condition was subjective and can be investigated in further experiments. Therefore we rely on the assumption that the obtained stimulation parameters are partially influenced by the natural activation of the quadriceps muscles during stance.

ILC control of FES has been recently proposed for position control of ankle [62] and hybrid FES-robot control of knee [52] combined with the Lokomat. In both cases, ILC controls the entire gait phase, as it is timely-defined by the fixed step cadence of Lokomat. ILC control is limited in Kinesis to the swing phase, with smooth and continuous transitions between gait phases. In [52], the control task is similar to Kinesis, where the interaction torque is minimized by the stimulation. Results in both cases are similar, although in [52] ILC converges in approximately 15 cycles whilst in Kinesis ranges from 5 to 10 gait cycles in average. This can be attributed to our non-conservative convergence criteria. We chose 5% of relative change in NILC as convergence criterion in order to avoid muscle fatigue with further iterations that are not monitored by the MFE. Additional iterations would only give little improvements in muscle force production but they would contribute to generating muscle fatigue.

Kinesis MFE allows not only for estimation of the effects of muscle fatigue but also stimulation performance within a learning scheme, by continuously monitoring generated torques. Results showed that a reduction in average of 30-40% of the first TTI within the first ILC iteration, which is directly related to stimulation performance. In addition, estimation of muscle fatigue and continuous monitoring of TTI allows for a robust management of muscle performance, implementing novel fatigue management strategies in hybrid neuroprosthesis (e.g. turning off the stimulation when the muscle is exhausted). In the reported experiences, we did not observed muscle exhaustion, which would be the case when TTI increases to first step TTI, where no stimulation is applied.

Kinesis is, to our best knowledge, the first ambulatory hybrid rehabilitation exoskeleton with stiffness control of knee trajectory. The cooperative control approach takes advantage of stiffness control in *monitoring state*, increasing the compliance of the robotic exoskeleton, balancing its assistance with the muscular force production, in a *reactive* version of the Assist-As-Needed concept. Experimental results have shown that Kinesis have reduced its stiffness during the swing phase to a minimum of 0 N ·m/deg. This decrease is shown to be correlated with TTI, therefore the cooperative controller effectively balances the robotic and neuroprosthetic power sources. During stance, Kinesis allows for a certain degree of knee motion, similar to [63]. However, specific analysis of stance phase was not undertaken, therefore we cannot extend the results from [63] to our approach.

Imposing both a kinematic and a time defined pattern on the patient is one of the limitations of Kinesis exoskeleton control. Further developments would include a more adaptable kinematic pattern to increase the cooperation between Kinesis and the residual abilities of the user. Surface electrical stimulation, although closed-loop modulated, is still not achieving a physiological activation pattern. Muscle activation in healthy conditions have several characteristics that are not synthesized with this approach, as muscle co-contractions and synergic activations, that contrast with the mechanistic approach implemented here. Further developments for rehabilitation purposes would include more bio-inspired stimulation controllers [64].

Optimal balance between neuroprosthetic and robotic actuation has been proposed in several works, but only results from simulation have been published [22,23]. These control approaches rely on accurate models of the neuromuscular system, currently a subject of major attention. Despite the theoretical effort done in those proposals, control and interaction with biological structures is still a challenging task, and more research on the areas of muscle modeling, physical human-robot interaction and control of hybrid exoskeletons is needed to design control strategies that optimally distribute the neuroprosthetic, robotic and user contribution to movement. In our approach, the correlation between the energy delivered by the robotic exoskeleton and TTI verifies that Kinesis cooperative control balances neuroprosthetic and robotic contributions. However this balance, although effective, cannot be demonstrated as optimal. Further studies should be conducted with Kinesis in order to investigate the performance of the cooperative control approach in comparison with current control approaches (i.e. position control of walking, automated robotic gait training).

The study with healthy volunteers presented in this article aimed to verify the hypothesis underlying the hybrid control approach and also testing the functional performance of Kinesis when used by humans. In addition, we aimed to test the protocol to be used with SCI patients. Walking velocity obtained in this experiment (0.44 ± 0.14 m/sec., Figure 9) is in line with previously published data of walking with passive orthosis (0.34 m/sec. for a reciprocating gait orthosis and 0.24 for the Wearable Orthosis [65]; 0.14 for a isocentric reciprocating gait orthosis [66]), and hybrid orthosis (0.14 to 0.45 for a hybrid reciprocating gait orthosis [67]). These data are still far from normative data regarding walking ability of people with SCI: 1.37 m/sec for the 10mWT [68]. Nevertheless, operation of Kinesis needs to trigger the step whenever the user is stable and ready to take it. This leads to a semi-automatic walking pattern that significatively reduces walking velocity, but provides safe operation to the patient. Questionnaires scores were included here in order to have information of healthy users perception. A general limitation arises with testing with healthy users, as they functionally behave different from people with SCI. Testing with healthy volunteers must be done prior to patient testing in order to ensure system stability and integrity, and refine control methods. In our experiments the healthy users were instructed to functionally behave similarly to impaired users, but we cannot ensure to what extent this was actually achieved.

Translation of the approach presented in this article to SCI patients can be challenging due to several factors. Among them, muscle atrophy and/or altered sensory perception can prevent from applying the stimulation and compensatory walking actions not compatible with the walking technique foreseen to be used with Kinesis, can hamper the use of a hybrid system by people with SCI. It can be noticed that setup time and complexity of the neuroprosthetic solution may represent additional time burden if compared with passive orthoses or robotic exoskeletons. Further improvements and hardware optimizations will be required to investigate the benefits of the hybrid approach with regard to its usability for daily use in clinical environments.

## Conclusion

A cooperative control strategy for a hybrid exoskeleton designed to deliver overground hybrid walking therapy with fatigue management has been presented, demonstrating its ability to balance the stimulation and robotic actuation, reflected in the correlation between leg and exoskeleton interaction. Closed-loop control of FES allows to manage changes in muscle performance and gait phase. This proposal overcomes several disadvantages related to FES control of movement: muscle fatigue is estimated through muscle performance and managed by closed-loop control of FES, and trajectory control through a compliant actuation of the exoskeleton. From these results, a clinical validation study with SCI target population will be completed in the National Hospital for Spinal Cord Injury (Toledo, Spain).

## Endnotes

^a^ As explained in section *Cooperative approach*, the FSM of Kinesis is comprised by two FSM operating in parallel: one in the time domain (t-FSM) and another operating in the cycle domain (c-FSM) (Figure 3).

^b^ The actual change on stimulation configuration is out of the scope of this article.

## Competing interests

The authors declare that does not have significant competing financial, professional or personal interests that might have influenced the performance or presentation of the work described in this manuscript.

## Authors’ contributions

AJA was responsible for Kinesis design and control approach proposal, and JCM and JLP supervised the work. Study design was done by AJA, JCM and AGA. Analysis and data interpretation was done by AJA, JCM. AJA and JCM drafted the manuscript. AGA and JLP reviewed the draft and made substantial comments. AGA and JLP were responsible for funding. All authors have read and approved the final manuscript.
